# Reliability of mechanical ventilation during continuous chest compressions: a crossover study of transport ventilators in a human cadaver model of CPR

**DOI:** 10.1186/s13049-021-00921-2

**Published:** 2021-07-28

**Authors:** Simon Orlob, Johannes Wittig, Christoph Hobisch, Daniel Auinger, Gabriel Honnef, Tobias Fellinger, Robin Ristl, Otmar Schindler, Philipp Metnitz, Georg Feigl, Gerhard Prause

**Affiliations:** 1 Division of Anaesthesiology for Cardiovascular Surgery and Intensive Care Medicine, Department of Anaesthesiology and Intensive Care Medicine, Medical University of Graz, Auenbruggerplatz 29, 8036 Graz, Austria; 2 Institute for Emergency Medicine, University Hospital Schleswig-Holstein, Campus Kiel, Arnold-Heller-Straße 3, 24105 Kiel, Germany; 3 Medical University of Graz, Auenbruggerplatz 2, 8036 Graz, Austria; 4 Division of General Anaesthesiology, Emergency- and Intensive Care Medicine, Department of Anaesthesiology and Intensive Care Medicine, Medical University of Graz, Auenbruggerplatz 29, 8036 Graz, Austria; 5 Centre for Medical Statistics, Informatics and Intelligent Systems, Medical University of Vienna, Spitalgasse 23, 1090 Vienna, Austria; 6 Department of Internal and Respiratory Medicine, Intensive Care Unit Enzenbach, State Hospital Graz II, Hörgas 30, 8112 Gratwein, Austria; 7 Division of Macroscopic and Clinical Anatomy, Medical University of Graz, Harrachgasse 21, 8010 Graz, Austria; 8 Institute of Morphology and Clinical Anatomy, Faculty of Health/School of Medicine, Witten/Herdecke University, Witten, Germany

**Keywords:** Cardiac arrest, Artificial respiration, Ventilators, mechanical, Cardiopulmonary resuscitation, Tidal volume, Reversed airflow, Out-of-hospital cardiac arrest

## Abstract

**Background:**

Previous studies have stated that hyperventilation often occurs in cardiopulmonary resuscitation (CPR) mainly due to excessive ventilation frequencies, especially when a manual valve bag is used. Transport ventilators may provide mandatory ventilation with predetermined tidal volumes and without the risk of hyperventilation. Nonetheless, interactions between chest compressions and ventilations are likely to occur. We investigated whether transport ventilators can provide adequate alveolar ventilation during continuous chest compression in adult CPR.

**Methods:**

A three-period crossover study with three common transport ventilators in a cadaver model of CPR was carried out. The three ventilators ‘MEDUMAT Standard²’, ‘Oxylog 3000 plus’, and ‘Monnal T60’ represent three different interventions, providing volume-controlled continuous mandatory ventilation (VC-CMV) via an endotracheal tube with a tidal volume of 6 mL/kg predicted body weight. Proximal airflow was measured, and the net tidal volume was derived for each respiratory cycle. The deviation from the predetermined tidal volume was calculated and analysed. Several mixed linear models were calculated with the cadaver as a random factor and ventilator, height, sex, crossover period and incremental number of each ventilation within the period as covariates to evaluate differences between ventilators.

**Results:**

Overall median deviation of net tidal volume from predetermined tidal volume was − 21.2 % (IQR: 19.6, range: [− 87.9 %; 25.8 %]) corresponding to a tidal volume of 4.75 mL/kg predicted body weight (IQR: 1.2, range: [0.7; 7.6]). In a mixed linear model, the ventilator model, the crossover period, and the cadaver’s height were significant factors for decreased tidal volume. The estimated effects of tidal volume deviation for each ventilator were − 14.5 % [95 %-CI: −22.5; −6.5] (*p* = 0.0004) for ‘Monnal T60’, − 30.6 % [95 %-CI: −38.6; −22.6] (*p* < 0.0001) for ‘Oxylog 3000 plus’ and − 31.0 % [95 %-CI: −38.9; −23.0] (*p* < 0.0001) for ‘MEDUMAT Standard²’.

**Conclusions:**

All investigated transport ventilators were able to provide alveolar ventilation even though chest compressions considerably decreased tidal volumes. Our results support the concept of using ventilators to avoid excessive ventilatory rates in CPR. This experimental study suggests that healthcare professionals should carefully monitor actual tidal volumes to recognise the occurrence of hypoventilation during continuous chest compressions.

**Supplementary Information:**

The online version contains supplementary material available at 10.1186/s13049-021-00921-2.

## Background

Sudden cardiac arrest is the third leading cause of death in Europe [1]. Out-of-hospital cardiac arrest (OHCA) has an annual incidence of approximately 89 per 100,000 inhabitants, resulting in more than 400,000 resuscitation attempts by emergency medical services every year in Europe and an overall survival rate of roughly 10 % [2, 3]. OHCA itself is a clinical condition that can be caused by several aetiologies. The predominant cause is of cardiac origin with underlying coronary disease. Cessation of organised cardiac contractions immediately leads to collapse of circulation with global ischemia, hypoxia, and global cell death.

To restore spontaneous circulation, cardiopulmonary resuscitation (CPR) strives to provide minimal perfusion with subsequent oxygen delivery to cells, predominantly of the heart and brain. Therefore, chest compressions and artificial ventilation have been a bundle of care in modern cardiac arrest treatment [4].

In recent years the role of ventilation has been comprehensively discussed [5, 6]. A major caveat in this debate has been the risk of hyperventilation in CPR [7]. As such, previous studies have posed that hyperventilation occurs commonly in CPR, mainly due to excessive ventilation frequencies, especially when a manual valve bag is used [8]. Nevertheless, apart from ventilation frequencies, actual tidal volumes (Vt) and minute volumes have been measured rarely in clinical CPR [8–11]. Ventilation during CPR can be synchronised with a compression-ventilation ratio of 30:2, or asynchronous with ventilations during continuous chest compressions [12]. While the synchronous ventilation strategy limits excessive manual ventilation frequencies, asynchronous manual ventilation does not. Still, continuous chest compressions may provide increased hemodynamic benefits regarding coronary perfusion pressure and limit no-flow time [13, 14]. Therefore, continuous chest compressions are recommended by international guidelines once the airway is secured [15].

Nevertheless, chest compressions are a counteracting force to positive pressure ventilation and may limit inspiratory volumes [16]. The risk of hypoventilation in CPR has been recently illustrated by Duchatelet et al. [11]. According to their study, chest compressions can impair tidal volumes, resulting in dead space ventilation without sufficient gas exchange.

Usage of mechanical ventilation in CPR might be a valid strategy to limit the respiratory rate and prevent tachy-ventilation during continuous chest compressions [6]. Whether common portable ventilators can provide relevant alveolar ventilation during continuous chest compressions is unknown. We sought to investigate the effect of continuous chest compressions on delivery of Vt, using transport ventilators in adults.

## Methods

A three-period crossover study with three common transport ventilators in a cadaver model of CPR was conducted. The three ventilators ‘MEDUMAT Standard²’ (WEINMANN Emergency Medical Technology GmbH + Co. KG, Hamburg, Germany), ‘Oxylog 3000 plus’ (Drägerwerk AG & Co. KGaA, Lübeck, Germany) and ‘Monnal T60’ (Air Liquide Medical Systems, Antony Cedex, France) represent three different interventions providing volume-controlled continuous mandatory ventilation (VC-CMV) under continuous automated chest compressions. The latter of the three ventilators was a turbine-driven ventilator. Preparation, measurements and interventions have been conducted following an exact study protocol [see flowchart, Additional file 1].

In 1992, Walter Thiel (Graz, Austria) developed an embalming process for human cadavers preserving their natural mechanical properties, known as Thiel’s method [17, 18]. Using this method, fixation is carried out over nine months by submersion of cadavers in basins of an embalming solution. Due to the close to in-vivo texture of the tissue, these cadavers are used in surgical training. More recently, they have also been used to study respiratory mechanics in models of resuscitation [19, 20].

All bodies were donated to the Chair of Macroscopic and Clinical Anatomy of the Medical University of Graz, under the strict rules of the anatomical donation program according to the Styrian burial law for scientific purposes. Hence, no additional approval by the local ethical board was required. For the present study, cadavers were randomly selected from the conservation basins and stored at ambient room temperature of 23 °C.

Cadavers were intubated orally by direct laryngoscopy. Tube positioning was verified by bronchoscopy (‘aScope™ 4 Broncho Large’, Ambu™, Ballerup, Denmark). Intrapulmonary fluid collections were suctioned through the bronchoscope’s working channel; the residue of the embalming process was removed by lavage.

A differential pressure sensor (‘DLVR-L60D’, All Sensors Corporation, Morgan Hill, California) was installed to the sideport of a heat and moisture exchange filter connected to the endotracheal tube. The sensor was zeroed to the atmospheric pressure. A mass flow meter (‘SFM3000’, Sensirion AG, Staefa, Switzerland) was placed distal to the filter, in line with the artificial airway.

Each sensor was connected to an individual small single-board computer (‘Raspberry Pi 3 B+’, Raspberry Pi Foundation, Cambridge, United Kingdom), recording raw signals. The sample rate of the flow meter was 200 and 500 Hz for the differential pressure sensor.

### Ventilatory strategy

The height of the cadaver was measured, and predicted body weight (PBW) was calculated using an adjusted Broca’s formula [21]. Ventilatory settings were chosen in accordance with the applicable guidelines [22, 23]. Throughout the experiment, volume-controlled ventilation was used. The target tidal volume was calculated as 6 mL/kg PBW. Tidal volumes were set to the closest possible values of the respective ventilator (Vt_set_). For sequences of chest compressions, a ventilatory frequency of 10/min, no positive end-expiratory pressure (PEEP), a pressure limit (P_max_) of 60 cmH_2_O with the shortest possible inspiratory period was used - being an inspiratory-expiratory-ratio of 1:5 for ‘Monnal T60’ and ‘Oxylog 3000 plus’, and 1:4 for ‘MEDUMAT Standard²’. The inspiratory period was set to the recommended inflation duration of 1 s.

### Chest compressions

Standardised chest compressions were performed using an automated chest compression piston device (‘Corpuls CPR’, GS Elektromedizinische Geräte G. Stemple GmbH, Kaufering, Germany). To avoid synchronisation of the chest compression phase and respiratory cycle, as given by a frequency of 100/min and 10/min, a chest compression frequency of 103/min and a compression depth of 5 cm was used.

### Study protocol

Respiratory mechanics were monitored by repeated pressure-volume curves (P/V loop) with an intensive care ventilator (‘HAMILTON-C6’, Hamilton Medical Inc., Bonaduz, Switzerland). Static compliance (C_stat_) was derived as the maximum slope of the inspiratory leg. Pneumothorax was ruled out by sonography after every two-minute cycle of chest compressions.

The initial aeration of the lung was carried out with the previously mentioned intensive care ventilator. Two quasi-static inflation manoeuvres were performed using a top pressure of 25 and 30 cmH_2_O. This was followed by a 15-minute sequence of ventilation (VC-CMV: Vt 6 mL/kg PBW, f 12/min; PEEP 5 cmH_2_O, I:E 1:2, P_max_ 40 cmH_2_O) [s. blue segment of flowchart, Additional file 1].

Lung and thoracic properties were assessed utilising three separate sequences, lasting two minutes each. During the first sequence only ventilation was provided, followed by chest compressions only, concluding with a combined sequence of chest compressions and ventilation [s. green segment of flowchart, Additional file 1].

The three transport ventilators were tested in a three-period crossover design. Each period consisted of two, two-minute-long segments of simulated CPR [s. yellow segment of flowchart, Additional file 1]. The possible permutations of ventilator orders were calculated in advance and randomly assigned to the individual cadaver using sealed envelopes.

In this publication, the results of the three-period crossover study are presented.

### Data processing & statistical analysis

**Fig. 1:**
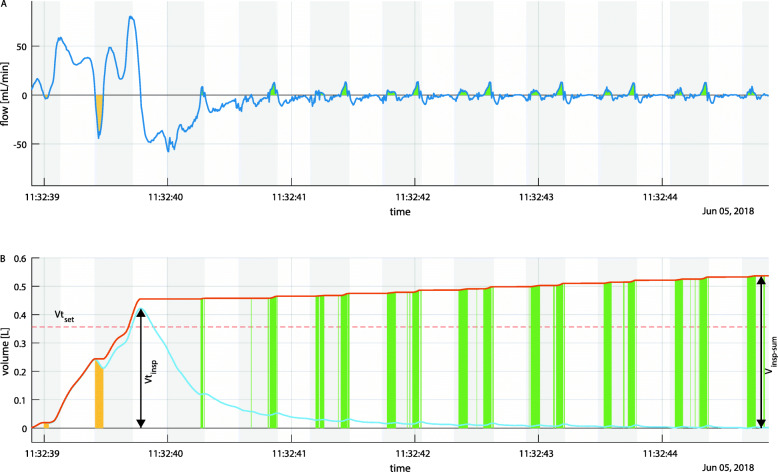
Flow (top) and derived volumes (below) of a single respiratory cycle under ongoing chest compressions. The light blue line represents net tidal volume, while the orange line represents cumulative inspiratory volume. Grey rectangles indicate intervals of chest compression. Yellow areas highlight reversed airflow in the inspiratory phase, while green areas highlight phases of expiratory reversed airflow. Vt_set_ is plotted as a broken red line. Arrows indicate derived values of volume

Raw signals were processed with ‘MATLAB’ (MathWorks, Natick, Massachusetts, United States). Tidal volumes were derived from the flow signal for every single respiratory cycle. Cumulative inspiratory volume was calculated as the volume of the total inward-directed airflow over the whole respiratory cycle (V_insp−sum_); thus, air movements due to chest compressions are also included. Inspiratory tidal volume was calculated as maximal inspiratory volume (Vt_insp_), therefore as the volume of net air inflow (s. Figure 1). Correspondingly, the same volumes were calculated for expiratory flow. Reverse airflow was calculated as the volume of opposite directed airflow to the respiratory phase - hence outward-directed airflow during the inspiratory phase and inward-directed airflow during the expiratory phase.

Missed inspiratory tidal volume (Vt_insp−missed_) was calculated as the deviation in percentage of inspiratory net tidal volume from Vt_set_. Peak flow was directly derived as inspiratory maxima for each respiratory cycle. Peak airway pressure was derived as maxima during the decompression phase for each respiratory cycle.

Two investigators (J.W. and S.O.) reviewed each ventilation separately and excluded ventilations without continuous chest compressions to the full depth of 5 cm in the inspiratory phase from further analysis.

Standard statistic software was used (‘IBM SPSS Statistics for Macintosh’, Version 26.0, IBM Corp., Armonk, New York, United States; ‘SAS 9.4’, SAS Institute, Cary, North Carolina, United States; ‘R 3.6.1’., R Development Core Team) for analysis. Parametric and non-parametric tests were conducted as indicated. Linear mixed models were calculated for Vt_insp−missed_, peak flow and peak airway pressure, each with the cadaver as the random factor and ventilator, height, sex, crossover period and incremental number of each ventilation within the period as covariates. The model specification allowed for different variances for each device. All data are reported as mean ± SD or median (IQR, [Minimum; Maximum]), as appropriate. Estimates are presented with their 95 %-confidence interval.

## Results

### Model characteristics

Six cadavers (three female, three male) were included in the study. Characteristics of cadavers and derived values are shown in Table 1. All measurements are available as raw data in a repository [24]. An executable plotting tool is available from another repository to review these ventilatory tracings [25].

**Table 1:** Characteristics and calculated ventilatory settings of individual cadavers

ID	sex	age [years]	height [cm]	PBW (calculated) [kg]	C_stat_ initial [mL/mbar]	Vt_opt_ (calculated 6 mL/kg) [mL]	Vt_set_ [mL]			Pneumothorax
							‘Monnal T60’	‘Oxylog 3000 plus’	‘MEDUMAT Standard²’	
G73	female	89	166	59.4	34.6	356	360	360	350	left, post 2nd period
G84	male	81	171	67.5	35.2	405	410	410	400	right, post 2nd period
G87	female	90	172	64.8	70.3	389	390	390	400	left, post 2nd period
G88	male	74	168	64.6	42.5	388	390	390	400	none
G74	male	83	166	62.7	32.5	376	380	380	400	none
G83	female	81	154	48.6	48.5	292	290	290	300	none

Median C_stat_ was 30.1 (11.9, [25; 51.5]) mL/mbar before chest compressions were started and increased with the first set of chest compressions up to 38.8 (19.8, [32.5; 70.3]) mL/mbar (*p* = 0.03). Further increase of static compliance was more subtle over the course of the experiment (2nd P/V loop: 43.1 (14.6, [31.3; 67.1]) mL/mbar, 3rd P/V loop: 48.5 (21.9, [34.8; 76.3]) mL/mbar, final P/V loop: 49.7 (22.2, [34.7; 70.8]) mL/mbar (*p* = 0.042) [s. Additional file 2].

### Tidal volumes

Within the 36 segments of simulated CPR, 757 single ventilations were recorded, of which 715 were valid for further analysis.

Inspiratory and expiratory tidal volumes differed in median by + 1.14 (8.68, [− 24.5; 35.4]) mL.

Overall, the delivered median Vt_insp_ was 274.8 (68.3 [47; 463.6]) mL. Normalized for body weight a median Vt_insp_ of 4.75 (1.2[0.7; 7.6]) mL/kg PBW.

Vt_insp−missed_ - meaning the percentage deviation of Vt_insp_ from the predetermined tidal volume (Vt_set_) - was − 21.2 (19.6, [− 87.9; 25.8]) %. For each ventilator Vt_insp−missed_ was in median − 8.3 (20.5, [–87.9; 25.8) % for ‘Monnal T60’, − 22.7 (22.1, [–70; − 12.3]) % for ‘Oxylog 3000 plus’ and − 31.5 (16.6, [–56.5; − 14.8]) % for ‘MEDUMAT Standard²’.

In a mixed linear model ventilator, crossover period and height were significant factors for Vt_insp−missed_ [s. Additional file 3]. The estimated population means of Vt_insp−missed_ for ventilator models were − 14.5 [95 %-CI: −22.5; −6.48] % (*p* = 0.0004) for ‘Monnal T60’, − 30.6 [95 %-CI: −38.6; −22.6] % (*p* < 0.0001) for ‘Oxylog 3000 plus’ and − 31 [95 %-CI: −38.9; −23] % (*p* < 0.0001) for ‘MEDUMAT Standard²’.

**Fig. 2:**
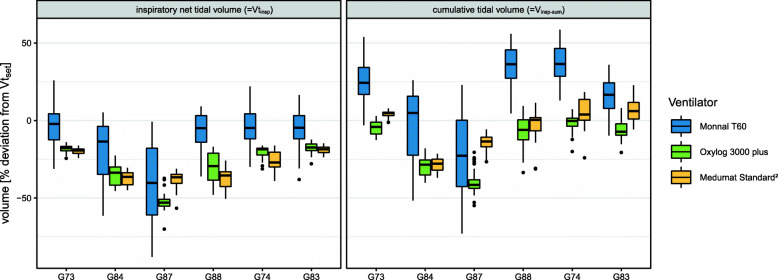
Deviation of net tidal volume from Vt_set_ for every single ventilation (=Vt_insp-missed_) as scatter plot separated for each ventilation crossover period. Colour represents the ventilator model. Marker shape represents individual cadavers. Trend lines are plotted for each two-minute-long segment of CPR and each particular cadaver separately

Cumulative inspiratory volume (V_insp−sum_) - meaning the total volume of inward-directed airflow over a single respiratory cycle - deviated from predetermined volume in median by + 20.1 (29.4, [− 72.9; 58.6]) % for ‘Monnal T60’, − 9.1 (25.6, [− 54.8; 9.4]) % for ‘Oxylog 3000 plus’ and + 0.4 (19.3, [− 36.9; 22.7]) % for ‘MEDUMAT Standard²’ (s. Figure 2).

Inspirations and expirations were fragmented by chest compressions. Per respiratory cycle of reversed airflow episodes, exceeding 1 mL of volume, occurred in median 1 (0, [0; 2]) time in inspiratory phase and 7 (1, [0; 9]) times in expiratory phase. Reversed airflow resulted in a median volume of 0.95 (0.96, [0.05; 2.87]) mL/kg PBW over the whole respiratory cycle, of which in median 0.05 (0.1, [0; 0.7]) mL/kg PBW occurred during inspiration and 0.87 (0.79 [0; 2.48]) mL/kg PBW during expiration. Maximal volume of a single episode of reversed airflow was 0.7 mL/kg PBW, corresponding to 45.6 mL.

### Flow and airway pressure

Median peak flow was 68.5 (23.6, [ 20.7; 93]) L/min, 44.4 (11, [27.9; 57.9]) L/min, 43.5 (8.1, [31.2; 54,9]) L/min for ‘Monnal T60’, ‘Oxylog 3000 plus’ and ‘MEDUMAT Standard²’, respectively. In a mixed linear model, we found that the differences of estimated effects by ventilators on peak flow between ‘Oxylog 3000 plus’ and ‘Monnal T60’ as well as between ‘MEDUMAT Standard²’ and ‘Monnal T60’ were significant (*p* < 0.001).

The median peak airway pressure during decompression phase was 46.6 (10.7, [30.2; 64.4]) mbar for ‘Monnal T60’, 55.5 (12.6, [27.9; 66]) mbar ‘Oxylog 3000 plus’ and 48.5 (14.4, [27; 73.3]) mbar Medumat Standard^2^. According to the linear mixed model, the differences between the estimated effects of ventilator models on airway pressure were significant (*p* < 0.001).

## Discussion

Our main finding is that all ventilators were able to provide tidal volumes exceeding anatomical deadspace under ongoing chest compressions with volume-controlled mandatory ventilation and a ventilatory frequency of 10/min. Thereby, all ventilators were able to deliver tidal volumes required for alveolar ventilation and gas exchange.

However, we also found all tested ventilators to provide substantially smaller tidal volumes than preset. Interestingly, the extent to which the tidal volumes were diminished was dependent on the ventilator model. While we found the turbine-driven ventilator to perform best, both pneumatic ventilators missed the preset tidal volume by almost a third. This compares to the results of a manikin study [16] and corresponds to the suspicion of hypoventilation in our previous clinical studies of blood gases in OHCA [26, 27]. These found that contrary to the common perception of hyperventilation in CPR, hypocapnia leading to alkalosis was not observed at all. Instead, most patients were found to be hypercapnic.

Oxygenation and decarboxylation are perceived as cornerstones of resuscitation efforts. Excessive hyperventilation has been shown to have detrimental effects on haemodynamics and resuscitation outcomes in an animal study [7]. In a trial with clinically more realistic tidal volumes, those results were not reproducible [28]. It is to be noted that the tidal volumes used in our experiment were substantially smaller than those used by Aufderheide and Gazmuri [7, 28, 29].

**Fig. 3:**
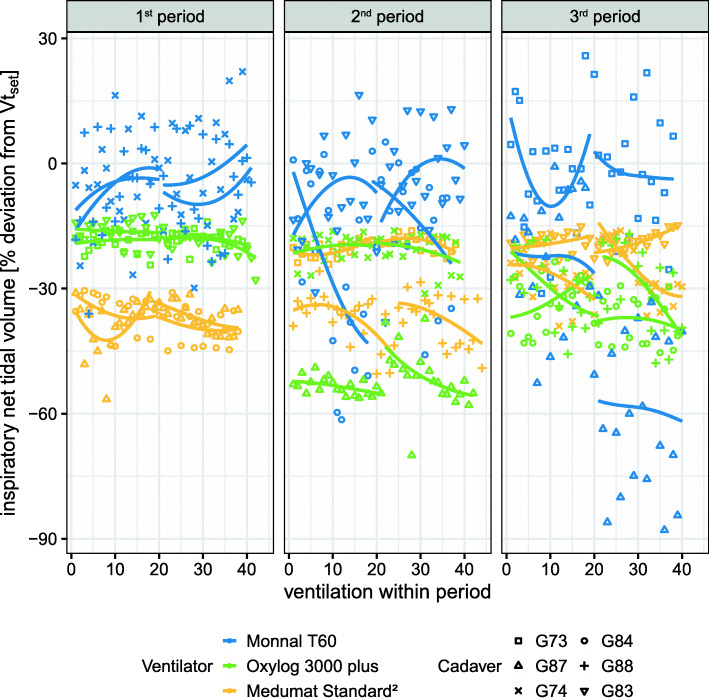
Deviation of the net (left) and cumulative (right) tidal volume from Vt_set_ as boxplot for each ventilator model grouped by individual cadavers. Colour represents the ventilator model. Note that due to standardization of the y-axis, Vt_insp_ resembles Vt_insp-missed_

We observed a periodical overshoot in net tidal volume in the turbine-driven ventilator, which indicates an internal control mechanism to compensate for diminished tidal volumes (s. Figure 3). This is a clinically relevant finding, as it implies that ventilators - although operating in the same mode - behave substantially different under ongoing chest compressions. The higher peak flow of the turbine-driven ventilator may suggest this device can deliver inspiratory flow more instantaneously during decompressions with less peak airway pressure.

We used endotracheal intubation as the airway gold standard with continuous chest compressions as recommended by the current guidelines [30]. The respiratory system was sealed, without a difference between inspiratory and expiratory tidal volumes. Clinically the focus should be on expiratory volumes, as they characterise alveolar ventilation more reliably due to possible leakage. For statistical analysis, we used the inspiratory volumes to quantify ventilation, as expiratory tidal volumes are influenced more by volatile changes of respiratory resting position due to chest compressions.

Chest compressions fragment ventilation through the occurrence of opposite directed airflow, known as reversed airflow. Compared to the observations by Duchatelet [11], we observed smaller volumes of reversed airflow and even episodes of opposite directed airflow in the expiratory phase. None of our observed reversed airflow episodes exceeded anatomical dead space. Thus, reversed airflow did not contribute to alveolar ventilation in our experiment but explains the difference between cumulative and net tidal volume. The distinction between cumulative and net tidal volumes is relevant. Total inspiratory airflow (V_insp−sum_) might suggest sufficient ventilation, while alveolar ventilation could be substantially less or even insufficient. Since we did not record displayed volumes of the ventilators, we cannot report the accuracy of their internal measurements and calculations. However, our results emphasise that the internal algorithm of the ventilators to calculate tidal volumes in CPR is of importance. Furthermore, our observations suggest that ventilatory volumes in CPR need to be reported and discussed in a more differentiated manner. Due to leakage and reversed airflow several varying tidal volumes can be calculated for a single ventilation. This should be acknowledged in clinical research to grant comparability of results, but also in clinical practice as only volumes contributing to gas exchange are meaningful.

Usage of human cadavers to study airflow phenomena might be advantageous over animal models as animals have substantial differences in the airway and thoracic configuration [31]. However, cadaver models are not suitable to observe haemodynamics, gas exchange or metabolic processes during resuscitation. In our experiment, static compliance was comparable to those observed previously in Thiel-embalmed cadavers [19] and immediately after termination of CPR [32].

The embalming process enriches the corpses with fluid. This might have contributed to the effect of increasing compliance with the application of chest compressions, possibly forcing fluid out of the parenchyma. Although the observed changes of static compliance were within a clinically plausible range, this might indicate deterioration of the model over time. Breakdown of endothelial function and the fluid load of the corpse are substantial limitations of this model.

Statistically, we compensated for deterioration effects over the course of the experiment. Due to the small sample size, we could not compensate for any carry-over effects in this crossover study. We have no comprehensive medical history of the body donors; therefore, pre-existing pulmonary conditions might have had an impact on cadaver-specific properties. This was considered in our statistical modelling with the cadavers as a random factor.

We used volume-controlled ventilation, as chest compressions increase airway pressure and thereby disturb pressure-controlled ventilation. This procedure is also recommended by recent guidelines [33]. Volume-controlled mode is standard in all ventilators but is more prone to cause barotrauma. When airway pressure exceeds P_max_, the expiratory valve is opened to reduce airway pressure. In CPR, peak airway pressure occurs during the compression phase. It has to be emphasised that this is the result of an extrapulmonary pressure increase and is as such not a cause for barotrauma per se, as transpulmonary pressure is not elevated [34]. In the present paper we are reporting peak airway pressure during the relaxation phase of chest compressions.

We chose a ventilatory strategy in accordance with the previous guidelines [22, 23]. These ventilatory settings also meet the 2021 guideline recommendations [30, 33], especially regarding the ventilatory mode, P_max,_ and ventilatory frequency. Even more, our approach lines up well with recently published ventilatory strategies for CPR [35, 36]. Nevertheless, we chose a conservative tidal volume of 6 mL/kg PBW, which is at the lower spectrum of the recommended 6–7 ml/kg PBW in the 2015 guidelines [22] and 2 ml/kg PBW less than recommended in the “Six-dial Strategy” [36]. Equally conservative, we decided to not use PEEP, although this is a controversial topic [37, 38]. While our settings avoided high airway pressures due to large tidal volumes and PEEP, both may have contributed to airway closure and atelectasis.

During CPR, atelectasis is common in dorsal lung areas with cyclic recruitment [39]. The increased ventilation in ventral lung areas implies the risk for volutrauma in those areas, while atelectotrauma occurs in the dorsal regions. As per protocol, we monitored for pneumothoraces. Those found by sonography were minor, did not reduce lung compliance and resulted in no air discharge upon thoracostomy at the end of the experiment.

In CPR, a severe ventilation-perfusion mismatch is present. Yet finding optimal ventilatory strategies is still an unsolved problem. Capnography as a sole concentration measurement of carbon dioxide is influenced by pulmonary blood flow and cannot quantify ventilation alone [40]. As in the present study, measurement of expiratory tidal volumes should become a clinical standard, as already demanded by Ornato et al. in 1983 [9]. Recent advances in technology made this readily available for manual ventilation as well [11, 41]. Furthermore, the nexus of capnography with tidal volumes as volumetric capnography might be a field of future developments in individualised, goal-directed CPR [42, 43].

## Conclusions

Our findings support the concept of using transport ventilators to limit the ventilation rate. Nevertheless, we also observed ventilations with severely low tidal volumes. Therefore, healthcare providers should closely monitor expiratory tidal volumes when using mechanical ventilation during continuous chest compressions without relying on preset values.

Due to reversed airflow, ventilations are fragmented in CPR, with a significant portion of airflow presumably not contributing to alveolar ventilation. Therefore, tidal volumes have to be considered in a much more differentiated way under ongoing chest compressions in clinical research to grant comparability of results, but also in clinical practice as only volumes contributing to gas exchange are meaningful. Future clinical studies are needed to confirm our findings.

## Supplementary Information

**Additional file 1. ** Experiment flowchart. Colour represents the phase of the experiment. Detailed ventilator and chest compression settings are given within the text balloons. Note that the yellow segment illustrates the crossover study, and the iterative workflow is only provided for the first period in detail.

**Additional file 2. ** Repetitive pressure-volume loops over the course of the experiment for each cadaver obtained by quasi-static inflation-deflation manoeuvres. Colour represents the chronological order of P/V loops.

**Additional file 3. ** Contrasts and coefficients of the three presented mixed linear models

## Data Availability

The datasets generated and analysed during the current study are available in the Data Mendeley repository, 10.17632/vh4tdsscns.1. An interactive visualisation tool to review the data is available from Data Mendeley repository, 10.17632/43h7zzp67k.3 (operating system: Windows; other requirements: MATLAB Runtime; license: CC BY 4.0).

## Abbreviations

95%-CI: 95 % confidence interval
aADCO_2_: Arterio-alveolar carbon dioxide gradient
CPR: Cardiopulmonary resuscitation
C_stat_: Static compliance
IQR: Interquartile range
OHCA: Out-of-hospital cardiac arrest
P/V loop: Pressure-volume loop
PBW: Predicted body weight
PEEP: Positive end-expiratory pressure
P_max_: Pressure limit
VC-CMV: Volume controlled continuous mandatory ventilation
V_insp−sum_: Cumulative inspiratory volume (volume of the total inward-directed airflow for a respiratory cycle)
Vt: Tidal volume
Vt_insp_: Inspiratory net tidal volume
Vt_insp−missed_: Missed inspiratory tidal volume (difference of Vt_insp_ and Vt_set_)
Vt_set_: Preset tidal volume
