# Supplementary material for: Reliability of mechanical ventilation during continuous chest compressions: a crossover study of transport ventilators in a human cadaver model of CPR
Source: Scand J Trauma Resusc Emerg Med. 2021 Jul 28;29:102. doi: 10.1186/s13049-021-00921-2 (PMC8316711; doi:10.1186/s13049-021-00921-2)
Supplement: Supplementary file 1 — Additional file 1. Experiment flowchart. Colour represents the phase of the experiment. Detailed ventilator and chest compression settings are given within the text balloons. Note that the yellow segment illustrates the crossover study, and the iterative workflow is only provided for the first period in detail. [file 13049_2021_921_MOESM1_ESM.pdf]

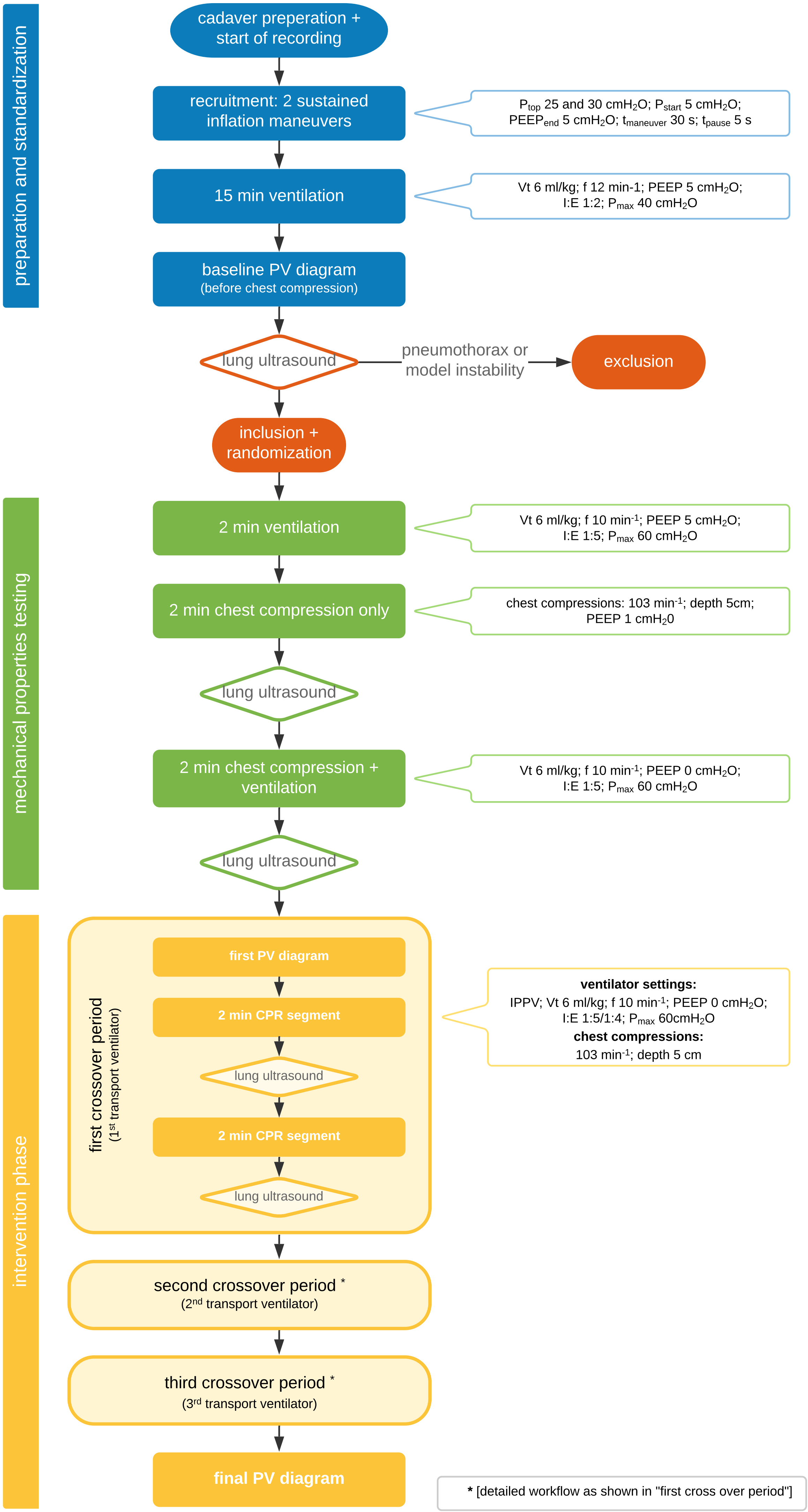

preparation and standardization
cadaver preperation + start of recording
recruitment: 2 sustained inflation maneuvers
P_{top} 25 and 30 cmH_2O; P_{start} 5 cmH_2O; $PEEP_{end}$ 5 cmH_2O; $t_{maneuver}$ 30 s; t_{pause} 5 s
15 min ventilation
Vt 6 ml/kg; f 12 min-1; PEEP 5 cmH_2O; I:E 1:2; P_{max} 40 cmH_2O
baseline PV diagram
(before chest compression)
lung ultrasound
pneumothorax or model instability
exclusion
inclusion + randomization
mechanical properties testing
2 min ventilation
Vt 6 ml/kg; f 10 min^{-1}; PEEP 5 cmH_2O; I:E 1:5; P_{max} 60 cmH_2O
2 min chest compression only
chest compressions: 103 min^{-1}; depth 5cm; PEEP 1 cmH_20
lung ultrasound
2 min chest compression + ventilation
Vt 6 ml/kg; f 10 min^{-1}; PEEP 0 cmH_2O; I:E 1:5; P_{max} 60 cmH_2O
lung ultrasound
intervention phase
first crossover period
(1st transport ventilator)
first PV diagram
2 min CPR segment
lung ultrasound
2 min CPR segment
lung ultrasound
ventilator settings:
IPPV; Vt 6 ml/kg; f 10 min^{-1}; PEEP 0 cmH_2O; I:E 1:5/1:4; P_{max} 60cmH_2O
chest compressions:
103 min^{-1}; depth 5 cm
second crossover period *
(2nd transport ventilator)
third crossover period *
(3rd transport ventilator)
final PV diagram
* [detailed workflow as shown in "first cross over period"]
