# Supplementary figures and images for: Reliability of mechanical ventilation during continuous chest compressions: a crossover study of transport ventilators in a human cadaver model of CPR
Source: Scand J Trauma Resusc Emerg Med. 2021 Jul 28;29:102. doi: 10.1186/s13049-021-00921-2 (PMC8316711; doi:10.1186/s13049-021-00921-2)

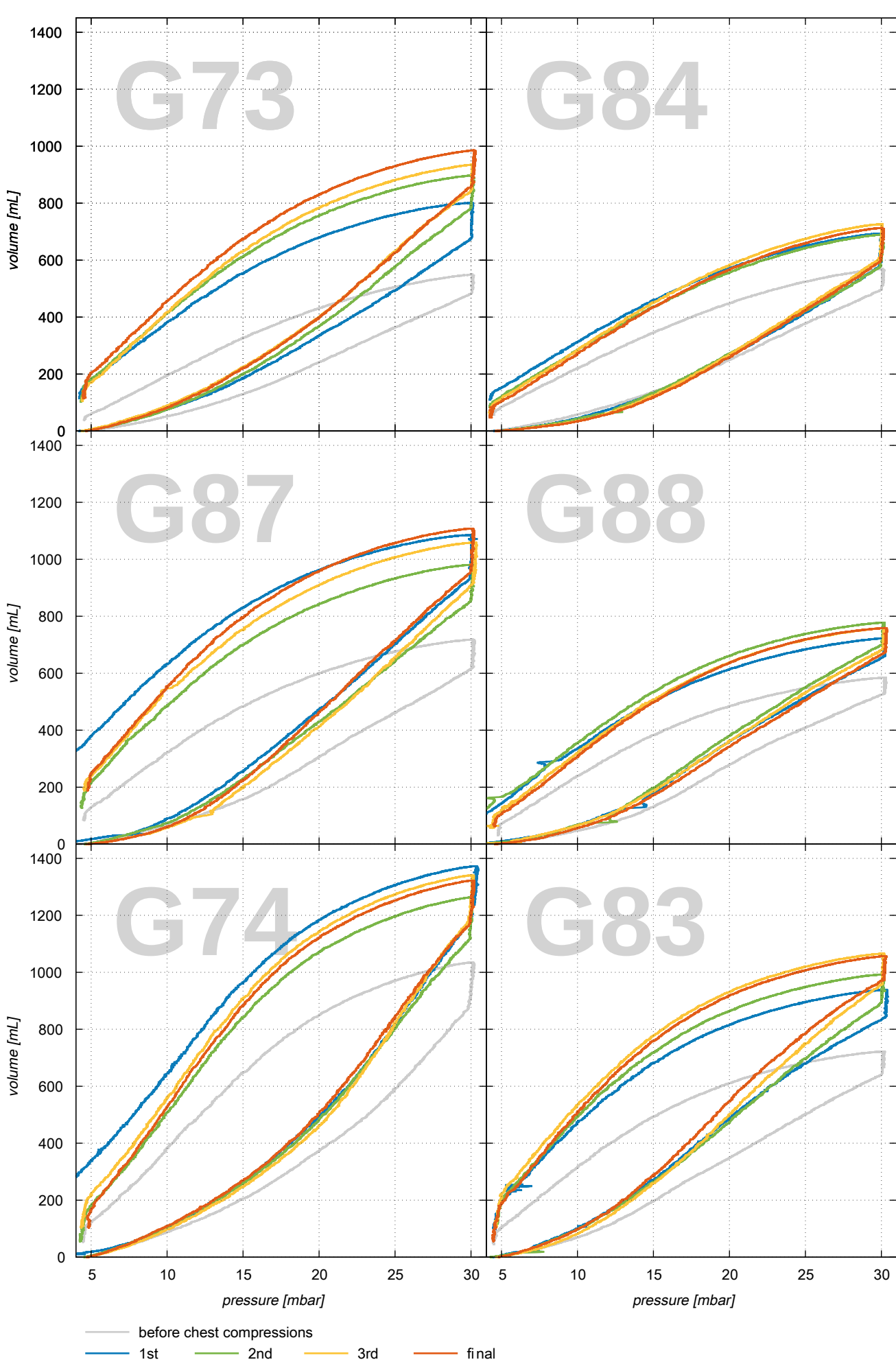

Supplement: Supplementary file 2 — Additional file 2. Repetitive pressure-volume loops over the course of the experiment for each cadaver obtained by quasi-static inflation-deflation manoeuvres. Colour represents the chronological order of P/V loops. [file 13049_2021_921_MOESM2_ESM.pdf]
