# Supplementary material for: Reliability of mechanical ventilation during continuous chest compressions: a crossover study of transport ventilators in a human cadaver model of CPR
Source: Scand J Trauma Resusc Emerg Med. 2021 Jul 28;29:102. doi: 10.1186/s13049-021-00921-2 (PMC8316711; doi:10.1186/s13049-021-00921-2)
Supplement: Supplementary file 3 — Additional file 3. Contrasts and coefficients of the three presented mixed linear models [file 13049_2021_921_MOESM3_ESM.pdf]

Supplemental Digital Content 4 tables with contrasts and coefficients of the three presented mixed linear models

1st linear mixed model for influence of ventilator model on $Vt_{insp\text{-}missed}$

**ventilator model**	**estimate**	**lower CI**	**Upper CI**	**t-value**	**p-value**
MonnalT60	-14,487	-22,495	-6,480	-3,550	0.0004
Oxylog3000	-30,557	-38,556	-22,558	-7,500	<.0001
MedumatStandard	-30,956	-38,942	-22,970	-7,610	<.0001

Supplement Table 1 estimated population means for the ventilator model, statistical model on missed inspiratory tidal volume

effect	level	estimate	degress of freedom	std. error	t-value	p-value
intercept		234,360	4	122,020	1,920	0.1272
ventilator model	MonnalT60	16,469	677	0,978	16,840	<.0001
ventilator model	Oxylog3000	0,399	677	0,972	0,410	0.6817
ventilator model	MedumatStandard	0,000				
segment of CPR	2	0,074	677	1,382	0,050	0.9576
segment of CPR	3	-7,124	677	1,384	-5,150	<.0001
segment of CPR	4	-3,901	677	1,381	-2,830	0.0049
segment of CPR	5	-2,255	677	1,383	-1,630	0.1035
segment of CPR	6	-8,886	677	1,383	-6,420	<.0001
segment of CPR	1	0,000				
breath within cross-over period	2	-4,010	677	2,876	-1,390	0.1638
breath within cross-over period	3	-4,857	677	2,876	-1,690	0.0918
breath within cross-over period	4	-2,570	677	2,876	-0,890	0.3719
breath within cross-over period	5	-3,771	677	2,876	-1,310	0.1903
breath within cross-over period	6	-5,128	677	2,876	-1,780	0.0751
breath within cross-over period	7	-2,124	677	2,876	-0,740	0.4606
breath within cross-over period	8	-3,221	677	2,876	-1,120	0.2632
breath within cross-over period	9	-4,836	677	2,876	-1,680	0.0932
breath within cross-over period	10	-0,017	677	2,988	-0,010	0.9955
breath within cross-over period	11	-4,894	677	2,892	-1,690	0.0910
breath within cross-over period	12	-8,032	677	2,876	-2,790	0.0054
breath within cross-over period	13	-6,054	677	2,892	-2,090	0.0367
breath within cross-over period	14	-3,530	677	2,876	-1,230	0.2202
breath within cross-over period	15	-6,078	677	2,876	-2,110	0.0350
breath within cross-over period	16	-6,867	677	2,876	-2,390	0.0172
breath within cross-over period	17	-2,673	677	2,876	-0,930	0.3531
breath within cross-over period	18	-4,152	677	2,876	-1,440	0.1493
breath within cross-over period	19	-6,520	677	2,876	-2,270	0.0237
breath within cross-over period	20	-3,629	677	3,086	-1,180	0.2399
breath within cross-over period	21	-8,597	677	3,297	-2,610	0.0093
breath within cross-over period	22	-9,273	677	6,603	-1,400	0.1606
breath within cross-over period	23	-12,125	677	7,887	-1,540	0.1247
breath within cross-over period	24	-21,329	677	10,927	-1,950	0.0514
breath within cross-over period	25	-14,729	677	10,927	-1,350	0.1782
breath within cross-over period	1	0,000				
height		-1,520	677	0,724	-2,100	0.0362
sex	female	-6,416	677	8,542	-0,750	0.4528
sex	male	0,000				

Supplement Table 2 fixed effects coefficients, statistical model on missed inspiratory tidal volume

2nd linear mixed model for influence of ventilator model on peak flow

contrast	estimate	std. error	p-value	lower CI	upper CI
Monnal T60	22,587	0,621	0,00000	21,132	24,043
Oxylog 3000	1,128	0,617	0,16042	-0,318	2,574
Monnal T60 – Oxylog 3000	21,459	0,617	0,00000	20,014	22,904

Supplement Table 3 contrasts with simultaneous confidence intervals, statistical model on peak flow

variable	level	coefficient
(intercept)		227,948
ventilator model	Monnal T60	16,468
ventilator model	Oxylog 3000	0,399
sex	male	6,416
height		-1,520
segment of CPR	2	0,074
segment of CPR	3	-7,124
segment of CPR	4	-3,901
segment of CPR	5	-2,255
segment of CPR	6	-8,886
breath within cross-over period	2	-4,010
breath within cross-over period	3	-4,857
breath within cross-over period	4	-2,570
breath within cross-over period	5	-3,771
breath within cross-over period	6	-5,128
breath within cross-over period	7	-2,124
breath within cross-over period	8	-3,221
breath within cross-over period	9	-4,836
breath within cross-over period	10	-0,017
breath within cross-over period	11	-4,894
breath within cross-over period	12	-8,032
breath within cross-over period	13	-6,054
breath within cross-over period	14	-3,530
breath within cross-over period	15	-6,078
breath within cross-over period	16	-6,867
breath within cross-over period	17	-2,673
breath within cross-over period	18	-4,152
breath within cross-over period	19	-6,520
breath within cross-over period	20	-3,629
breath within cross-over period	21	-8,597
breath within cross-over period	22	-9,273
breath within cross-over period	23	-12,125
breath within cross-over period	24	-21,329
breath within cross-over period	25	-14,729

Supplement Table 4 fixed effects coefficients, statistical model on peak flow

3rd linear mixed model for influence of ventilator model on peak airway pressure

contrast	estimate	std. error	p-value	lower CI	upper CI
Monnal T60	-9,166	0,338	0,00000	-9,957	-8,374
Oxylog 3000	1,445	0,336	0,00006	0,658	2,231
Monnal T60 – Oxylog 3000	-10,610	0,335	0,00000	-11,396	-9,824

Supplement Table 5 contrasts with simultaneous confidence intervals, statistical model on peak airway pressure

**variable**	**level**	**coefficient**
(intercept)		227,948
ventilator Model	Monnal T60	16,468
ventilator Model	Oxylog 3000	0,399
sex	male	6,416
height		-1,520
segment of CPR	2	0,074
segment of CPR	3	-7,124
segment of CPR	4	-3,901
segment of CPR	5	-2,255
segment of CPR	6	-8,886
breath within cross-over period	2	-4,010
breath within cross-over period	3	-4,857
breath within cross-over period	4	-2,570
breath within cross-over period	5	-3,771
breath within cross-over period	6	-5,128
breath within cross-over period	7	-2,124
breath within cross-over period	8	-3,221
breath within cross-over period	9	-4,836
breath within cross-over period	10	-0,017
breath within cross-over period	11	-4,894
breath within cross-over period	12	-8,032
breath within cross-over period	13	-6,054
breath within cross-over period	14	-3,530
breath within cross-over period	15	-6,078
breath within cross-over period	16	-6,867
breath within cross-over period	17	-2,673
breath within cross-over period	18	-4,152
breath within cross-over period	19	-6,520
breath within cross-over period	20	-3,629
breath within cross-over period	21	-8,597
breath within cross-over period	22	-9,273
breath within cross-over period	23	-12,125
breath within cross-over period	24	-21,329
breath within cross-over period	25	-14,729

Supplement Table 6 fixed effects coefficients, statistical model on peak airway pressure
